# Supplementary material for: A comparison of microbial community composition in two alpine springs in southern Nevada
Source: PLoS One. 2026 Feb 27;21(2):e0342925. doi: 10.1371/journal.pone.0342925 (PMC12948051; doi:10.1371/journal.pone.0342925)
Supplement: S1 Table — (DOCX) [file pone.0342925.s001.docx]

**S1 Table:** Characteristics of Harris Spring and Deer Creek Spring.

| **Parameters** | **Harris Spring** | **Deer Creek Spring** |
| --- | --- | --- |
| Latitude | 36.24052 | 36.30680 |
| Longitude | -115.54346 | -115.63574 |
| Elevation | 1,779 m | 2,786 m |
| Expression of Water Flow | Rheocrene | Rheocrene and Hillslope |
| Landscape Geology (Page et al. 2005) | Old alluvium (2.58 Ma - 0.0117 Ma) and intermediate fan alluvium (0.129 Ma) | Heavily fractured bedrock of the Antelope Valley Limestone, Goodwin Limestone of the Pogonip Group (538.8 Ma - 458.4 Ma), Narrow Canyon Formation, Crystal Pass Limestone, Guilmette Formation, Sultan Limestone, undivided (393.3 Ma - 346.7 Ma), and Monte Cristo Group (358.9 Ma - 323.2 Ma) |
| Landscape Ecology by Life Zones  (Tingley et al. 2008) | Upper Sonoran: shadscale, blackbrush, creosote bush, horsebush, lycium, jojoba, Joshua tree forest) | Canadian (Montane): Douglas fir, spruce, aspen |
| Dates of Data Acquisition | June 15, 2022  June 29, 2022  July 13, 2022 | June 13, 2022  June 27, 2022  July 11, 2022 |
| Water Temperature (°C) | 16.6 | 10.1 |
| pH | 7.84 | 8.16 |
| Electrical Conductivity (μS/cm) | 693 | 299 |
| Total Dissolved Solids (ppm) | 501 | 211 |
| Sodicity (ppt NaCl) | 0.35 | 0.15 |
| Alkalinity (ppm) | 281 | 180 |
| Dissolved Oxygen (ppm) | 7 | 11 |
