# Supplementary material for: A comparison of microbial community composition in two alpine springs in southern Nevada
Source: PLoS One. 2026 Feb 27;21(2):e0342925. doi: 10.1371/journal.pone.0342925 (PMC12948051; doi:10.1371/journal.pone.0342925)
Supplement: S2 Table — (DOCX) [file pone.0342925.s002.docx]

**S2 Table:** Water chemistry parameters by measurement date at Harris Spring and Deer Creek Spring.

| **Date** | **Water Temperature (°C)** | **pH** | **Conductivity (μS/cm)** | **Total Dissolved Solids (ppm)** | **Salinity (ppt NaCl)** | **Alkalinity (ppm CaCO_3_)** | **Dissolved Oxygen (ppm)** |
| --- | --- | --- | --- | --- | --- | --- | --- |
| **Harris Spring Upper** | | | | | | | |
| June 15 2022 | 14.4 | 7.43 | 732 | 543 | 0.37 | N/A | N/A |
| June 29 2022 | 15.2 | 7.45 | 686 | 491 | 0.34 | 330 | 6 |
| July 13 2022 | 15.6 | 7.60 | 670 | 485 | 0.35 | 380 | 5 |
| **Harris Spring Middle** | | | | | | | |
| June 15 2022 | 14.8 | 7.86 | 729 | 522 | 0.38 | 200 | 5 |
| June 29 2022 | 15.9 | 7.81 | 683 | 492 | 0.33 | 300 | 5 |
| July 13 2022 | 17.1 | 8.07 | 631 | 465 | 0.35 | 300 | 12 |
| **Harris Spring Lower** | | | | | | | |
| June 15 2022 | 20.0 | 8.13 | 711 | 519 | 0.37 | 175 | 6 |
| June 29 2022 | 18.9 | 8.15 | 674 | 478 | 0.34 | 310 | 7 |
| July 13 2022 | 17.9 | 8.27 | 666 | 476 | 0.33 | 340 | 10 |
| **Deer Creek Upper** | | | | | | | |
| June 13 2022 | 7.1 | 8.03 | 230 | 158 | 0.11 | 94 | 7 |
| June 27 2022 | 5.9 | 7.91 | 293 | 201 | 0.15 | N/A | 8 |
| July 11 2022 | 6.5 | 7.88 | 260 | 188 | 0.13 | N/A | N/A |
| **Deer Creek Middle** | | | | | | | |
| June 13 2022 | 8.7 | 8.38 | 244 | 175 | 0.12 | 115 | 7 |
| June 27 2022 | 12.4 | 8.35 | 279 | 199 | 0.14 | N/A | N/A |
| July 11 2022 | 11.2 | 8.32 | 311 | 219 | 0.15 | N/A | N/A |
| **Deer Creek Lower** | | | | | | | |
| June 13 2022 | 10.5 | 8.12 | 304 | 217 | 0.15 | 200 | 11 |
| June 27 2022 | 12.6 | 8.03 | 364 | 255 | 0.18 | 250 | 6 |
| July 11 2022 | 15.6 | 8.41 | 403 | 289 | 0.21 | 240 | 25 |
