# Supplementary material for: A comparison of microbial community composition in two alpine springs in southern Nevada
Source: PLoS One. 2026 Feb 27;21(2):e0342925. doi: 10.1371/journal.pone.0342925 (PMC12948051; doi:10.1371/journal.pone.0342925)
Supplement: S1 File — (DOCX) [file pone.0342925.s003.docx]

**S1 File:** FASTQ Files for Spring Mountains, Nevada.

<https://doi.org/10.6084/m9.figshare.30715514>
