# Supplementary material for: A comparison of microbial community composition in two alpine springs in southern Nevada
Source: PLoS One. 2026 Feb 27;21(2):e0342925. doi: 10.1371/journal.pone.0342925 (PMC12948051; doi:10.1371/journal.pone.0342925)
Supplement: Attachment — Submitted filename: Review PlusONE.docx [file pone.0342925.s004.docx]

**Abtract**

In lines 19- 20 the statement “The authors acquired, 20 transported, amplified, and extracted eDNA and measured water...” should be rewritten as eDNA was acquired, transported.....”. Remove “the Author” and the use of too many “and” in the sentence.

The abstract doesn’t represent the entire report. Results were not presented, the statistical observations were not indicated and the implications for the study were neither mentioned as a basis for the conclusion. For example in lines 22-23 Authors said “Results show that when compared to Harris Spring, Deer Creek Spring was colder, more alkaline, had lower conductivity and total dissolved solids/salinity concentrations”. Words like “lower”, “colder”, “more alkaline” are unstatistical and unscienific.

The abstract should thus be rewritten to represent the main report.

**Introduction**

The introduction is too lengthy. Authors should focus on providing a compelling narrative to justify the study and set a precedence for the research conducted.

The introduction should end with a clear statement of the research problem and the particular knowledge gap the study is aimed at filling.

The introduction contains tables from previous studies. This is strongly discouraged. Tables from other works may come in as discussions. The introduction section of article usually is void of tables and figures.

**Results**

The statement “Water temperature was higher at Harris Spring than Deer Creek Spring” in line 229 is indefinite as the actual temperature figures were not mentioned whereas succinct description is the results is main goal of this section.

Results have been presented in bullet points like power point presentation.

**References**

Some references are too old. All references older than 10 years should be replaced.
