# Supplementary material for: A comparison of microbial community composition in two alpine springs in southern Nevada
Source: PLoS One. 2026 Feb 27;21(2):e0342925. doi: 10.1371/journal.pone.0342925 (PMC12948051; doi:10.1371/journal.pone.0342925)
Supplement: Attachment — Submitted filename: Revisions Checklist and Reasoning.docx [file pone.0342925.s005.docx]

**RESPONSE TO EDITOR COMMENTS**

| **Suggested Revisions** | **Revisions Made by Authors** | **Reasoning** |
| --- | --- | --- |
| Can you please upload an additional copy of your revised manuscript that does not contain any tracked changes or highlighting as your main article file. This will be used in the production process if your manuscript is accepted.  Please amend the file type for the file showing your changes to Revised Manuscript w/tracked changes. | All suggested revisions have been completed. | Following editor’s requirements for proper article submission. |
| Please amend the title either on the online submission form or in your so that they are identical. | All suggested revisions have been completed. | Following editor’s requirements for proper article submission. |
| Please amend your manuscript to include a reference list. References must be placed at the end of the manuscript and numbered in the order that they appear in the text. | All suggested revisions have been completed. | Following editor’s requirements for proper article submission. |
| Please upload a Response to Reviewers letter which should include a point by point response to each of the points made by the Editor and / or Reviewers. (This should be uploaded as a 'Response to Reviewers' file type.) | All suggested revisions have been completed. | Following editor’s requirements for proper article submission. |

**ABSTRACT**

| **Suggested Revisions** | **Revisions Made by Authors** | **Reasoning** |
| --- | --- | --- |
| In lines 19- 20 the statement “The authors acquired, 20 transported, amplified, and extracted eDNA and measured water...” should be rewritten as eDNA was acquired, transported.....”. Remove “the Author” and the use of too many “and” in the sentence. | Sentence was modified in response to the suggestion. | Authors agree with the reviewer's comments. |
| The abstract doesn’t represent the entire report. Results were not presented, the statistical observations were not indicated and the implications for the study were neither mentioned as a basis for the conclusion. For example in lines 22-23 Authors said “Results show that when compared to Harris Spring, Deer Creek Spring was colder, more alkaline, had lower conductivity and total dissolved solids/salinity concentrations”. Words like “lower”, “colder”, “more alkaline” are unstatistical and unscienific. | Abstract has been rewritten to cover the scope of the manuscript. | Authors agree with the reviewer's comments. |
| The abstract should thus be rewritten to represent the main report. | Abstract has been rewritten to cover the scope of the manuscript. | Authors agree with the reviewer's comments. |
| Sky island is likely not a geographic term. | No revision made. | Sky island is a well-known scientific term used to describe isolated mountain ranges surrounded by significantly different lowland habitats and has been in use since at least the mid-20th century. |
| Mention how many sites per spring were sampled. | Added to the abstract. | Authors agree with the reviewer's comments. |
| What is your hypothesis? | A purpose of the study has been mentioned in the Abstract added three goals were added to the Introduction section. | Authors agree with the reviewer's comments. |
| What is the scientific importance/justification for the study? | The authors have included this information in the Abstract. Additionally, further discussions of ecosystem impact and conservation efforts have been elaborated in the Discussion section. | Authors agree with the reviewer's comments. |
| Write in past tense (throughout). | This change has been made. | Authors agree with the reviewer's comments. |
| The last sentence is an assumption. | This sentence has been removed and replaced with a clearer sentence that is not an assumption. | Authors agree with the reviewer's comments. |

**INTRODUCTION**

| **Suggested Revisions** | **Revisions Made by Authors** | **Reasoning** |
| --- | --- | --- |
| The introduction is too lengthy. Authors should focus on providing a compelling narrative to justify the study and set a precedence for the research conducted. | Length of the Introduction has been greatly reduced. All content considered “literature review” has been removed. Study site descriptions was moved to a new Methods section “Study Sites”. | The remaining text focuses on providing a compelling narrative to justify the study and set precedence for the research conducted. |
| The introduction should end with a clear statement of the research problem and the particular knowledge gap the study is aimed at filling. | This change has been made. A purpose and three goals have been clearly defined in the Introduction. | Authors agree with the reviewer's comments. |
| The introduction contains tables from previous studies. This is strongly discouraged. Tables from other works may come in as discussions. The introduction section of article usually is void of tables and figures. | Table 1 in the Introduction has been moved to Supplemental Section. Table is now named Table S1.  All subsequent tables have been renumbered. | The Introduction does not contain tables from previous studies. Table 1, in the Introduction, contains rows cited from previous studies. For example, landscape ecology by life zone (Tingley et al. 2008) and landscape geology (Page et al. 2005) are citations from which the information originates.  Regardless, Table 1 has been moved to the Supplemental Section as per reviewers’ request. All subsequent tables have been renumbered to account for removal of Table 1. |
| Reads like a book report. (Authors cite page numbers of others work). What is the relevance of this material to your study? | Length of the Introduction has been greatly reduced. All content considered “literature review” has been removed. | The “book report” nature of the Introduction was an attempt to provide a brief literature review of the subject matter in a journal with a broad scientific scope. The literature review text has been removed. The remaining text focuses on providing a compelling narrative to justify the study and set precedence for the research conducted. |
| What is your hypothesis and the justification for this study? | A purpose of the study has been mentioned in the Abstract added three goals were added to the Introduction section. | Authors understand the need for clarity regarding the purpose and goals of this exploratory study. The proper changes have been made. |
| Line 53-59. Define metrics, water chemistry, and microbiome.(Ln. 149 – be specific) | All requested terms have been defined. | Authors agree with the reviewer's comment. |
| Be more thorough about description of eDNA sources (Line 60). | Additional language has been added to be more descriptive. | Authors agree with the reviewer's comment. |
| Ln. 64. How can eDNA be in samples? | This phrase was removed. | This phrase was removed due to confusion. |
| Suggest making a table for background on eDNA applications with citing specific articles relevant only to your study. Lns 66-69 are not comprehensive. | List of background applications on eDNA has been removed. The authors did not create a table. | A list or table of background applications on eDNA will never be comprehensive. Thus, in the spirit of the reviewer’s comments, this list has been removed. |
| Ln. 74. Trending means what? | The word “trending” has been removed to avoid confusion. | “Trending” is a geologic term to describe the azimuth direction of the long axis of a geographic feature. The term has been removed to avoid confusion and to avoid over explanation in the text. |
| Ln 83. Sky island? | No revisions have been made to the inclusion of this geographic term. | A sky island is an isolated mountain range that is surrounded by a dramatically different lowland environment. |
| Ln. 91. That 90% figure cannot be correct. | Authors clarified this statement. | The reviewer is probably referring to the claim that “The Spring Mountains get their name from the over 300 springs that emerge and flow at all elevations of the mountain range and support upwards of 80% of terrestrial and riparian species.” Sada et al. (2005) claim that “springs provide resources for numerous terrestrial species; for example, approximately 80% of the terrestrial animal species in the Great Basin are facultative or obligate users of riparian systems.” |
| Ln. 101. “Wild” is inappropriate. | The term has been deleted. | Authors agree with the reviewer's comment. |
| Ln. 110. Only now is community composition discussed and although it is a focus for the survey, it seems like an afterthought in the way it is being present. Ln. | Changes have been made to both the Abstract and the Introduction to include discussions of microbial community composition. | Authors agree with the reviewer's comment. |
| Rheocrene and similar terms need definition and citation. | Rheocrene and similar terms have been defined. | Authors agree with the reviewer's comment. |
| There is little rationale for site selection along the Springs. | No changes made. | This is not accurate. Upper location at each spring was near the spring source (where groundwater emerges as surface water). Other two locations (middle and lower) were evenly spaced down the rheocrene channel to span lateral distance and elevation ranges. |
| Tables. Should be in supplement. Why data in the Intro? | Table 1 has been moved to Supplemental section and renamed Table S1. | Regardless, Table 1 has been moved to Supplemental Section as per reviewers’ request. All subsequent tables have been renumbered to account for removal of Table 1. |
| Ln. 152. Microorganisms can in fact travel by air – provide context and citations. | The final sentence of this paragraph has been deleted. | The original intent of this sentence was to stress how sky islands may allow for evolution of endemic species. This sentence was removed because it added confusion (i.e., microbial transport), but also because it was repetitive of previous sky island statements. |
| The introduction can be strengthened by linking spring ecosystem studies to broader hydrologic and climatic processes. For instance, hydropower and reservoir impacts on hydrologic cycles (Li et al., 2025; Huang et al., 2025) and global isotope datasets (Li et al., 2025) provide useful comparisons. | No changes made. | These references, although useful, are not quite relevant to the current study due to the scale of infrastructure projects (i.e., hydropower and reservoir impacts). Many springs in the southwestern United States are quite small –you could leap across the width of the spring. Spring conservation in the Spring Mountains includes efforts like limiting human and wild ungulate access by constructing fences. Small herds of donkeys, horses, or deer could trample and ruin spring ecosystems, and humans driving off road vehicles heavily alter streambed morphology. Thus, there is a misalignment in stream channel and discharge magnitude when relating these springs to publications discussing hydropower projects. |
| Kindly highlight the significance of study in last paragraph of Introduction. | The original final paragraph of the Introduction has been separated into two paragraphs. Some text has been removed and more text added. | These changes hopefully bolster the significance of the study. A study purpose has been clearly articulated. Goals have been added in a numbered list (in prose). |

**METHODS**

| **Suggested Revisions** | **Revisions Made by Authors** | **Reasoning** |
| --- | --- | --- |
| In your Methods section, please provide additional information regarding the permits you obtained for the work. Please ensure you have included the full name of the authority that approved the field site access and, if no permits were required, a brief statement explaining why. | No permits were required. This text and an explanation has been added to the eDNA Acquisition subsection in the Materials and Methods section. | Text added includes reasoning: “No spring water was collected. Permits were not required as both study sites were located on public lands, and neither water samples nor protected species were removed from the field sites.” |
| Throughout there is not enough information on instruments for manufacturer, sources, and details. | No revisions were made. | Manufacturer and/or brand name of all instruments and test kits are included in the manuscript. The authors did not link to any manufacturer website or provide manufacturer citations as some journals do not approve of this form of advertising. Please advise if journal wishes for authors to add this type of information. |
| Many details can be included in supplement. | Some material has been moved to the Supplemental section in response to reviewers comments. | Authors agree with the reviewer's comments. |
| Ln. 188. Water samples from which eDNA was derived were collected. Correct the wording, please. | The wording has been corrected. | Authors agree with the reviewer's comments. |
| Ln. 192. That high number (83 x) of filtering with a 60 mL syringe with a filter is not realistic or practical. The number of times is not a competition (see Ln. 193). Errors and contamination could be introduced. | This sentence has been removed. | Authors have made the change the reviewer requested. |
| Ln. 201. Cite the use of the -20C rather than -80C. | Clarification as to the use of the –20C freezer has been added. | Recent studies such as Mauvisseau et al. (2021) show that freezing in a –20C freezer as a preservation strategy yields good results when compared to other preservation strategies. It appears the literature shows a –80C freezer is not required to store filtered eDNA samples. |
| Ln. 215. Who did the work? Specify. | Addition of the phrase “...by a field crew supervised by the authors” | Added to clarify that the authors were present during data collect to oversee the process. |
| Methods should clarify whether “disturbance level” was quantified or based on qualitative observations. | Language was altered to better reflect qualitative observations of disturbance. |  |

**RESULTS**

| **Suggested Revisions** | **Revisions Made by Authors** | **Reasoning** |
| --- | --- | --- |
| The statement “Water temperature was higher at Harris Spring than Deer Creek Spring” in line 229 is indefinite as the actual temperature figures were not mentioned whereas succinct description is the results is main goal of this section. | Results and Discussion sections have been completely rewritten. | Authors agree with the reviewer's comments. |
| Results have been presented in bullet points like power point presentation. | Bullet points have been changed to prose. | Authors agree with the reviewer's comments. |
| Results. Present them, not generalities. | Results and Discussion sections have been completely rewritten. | Authors agree with the reviewer's comments. |
| Show the data in interesting way, then use the discussion to interpret the data. | Results and Discussion sections have been completely rewritten. | Authors agree with the reviewer's comments. |
| Ln. 244. Opportunistic measures should have been described in the methods, with that rationale. | Results and Discussion sections have been completely rewritten. Methods section has also been rewritten to clarify methodology. | Authors agree with the reviewer's comments. |
| The statement “Water temperature was higher at Harris Spring than Deer Creek Spring” in line 229 is indefinite as the actual temperature figures were not mentioned whereas succinct description is the results is main goal of this section. | Results and Discussion sections have been completely rewritten. | Authors agree with the reviewer's comments. |
| Results have been presented in bullet points like power point presentation. | Bullet points have been changed to prose. | Authors agree with the reviewer's comments. |
| ANOVA should be conducted to statistically compare the physicochemical parameters across the stations before a comparison can be made with declaration of the probability level. Authors compared activities around the catchment area and this is not the right approach. | Mixed-effects ANOVA was conducted for the aquatic chemistry data. | Authors agree with the reviewer's comments. |
| The geomorphology section could draw from case studies in other volcanic and fractured terrains (D Silva et al., 2024; Abegeja & Nedaw, 2024). This would highlight how fractured geology and landscape evolution influence spring chemistry. | The geomorphology content has mostly been either moved to the Supplemental section or removed. | These changes have been made in response to other reviewer comments suggesting the manuscript has too much of a literature review. |

**DISCUSSION**

| **Suggested Revisions** | **Revisions Made by Authors** | **Reasoning** |
| --- | --- | --- |
| Discuss your own data in relations to others. For example, Ln. 424. Did you measure UV or any other metric such as landcover that is a less subjective than an observation of tree species nearby. | Clarifying language has been added to the Discussion section to delineate which were measured (i.e., aquatic chemistry parameters) and which were observed (i.e., solar radiation, anthropogenic disturbance, and stream reemergence). The authors then discuss how further research into these relationships can aid in ongoing conservation efforts in the Spring Mountains. | Authors agree with the reviewer's comments. |
| The discussion mentions drought but could be deepened by linking with global precipitation extremes and warming–hydrology interactions (Zhang & Wu, 2025; Zhu et al., 2024). | The mention of drought has been removed as this parameter has not been fully explored by the authors. | Warming-hydrological interactions, although interesting, is not the focus of the Discussion section anymore. |
| The role of anthropogenic disturbance is well described. Linking this with applied remediation or bio-inspired solutions (Ma et al., 2025) could increase applied relevance. | Although Ma et al. (2025) is a great citation, the authors have added other recent citations to this Discussion topic. | The authors believe the other added citations are more relevant to the type of springs found in the Spring Mountains. |
| You may discuss, latest current work of this field with emphasis on microbial population of springs. | Additional references to recent publications have been added in the Discussion section, particularly when pontificating on environmental and anthropogenic variables that may impact microbial community composition differences between Deer Creek Spring and Harris Spring. | Authors agree with the reviewer's comments. |

**CONCLUSION**

| **Suggested Revisions** | **Revisions Made by Authors** | **Reasoning** |
| --- | --- | --- |
| Conclusion should explicitly highlight conservation and management implications for cold-water springs in arid mountain ecosystems. | These changes have been made. | Authors agree with the reviewer's comments. |
| Provide a robust conclusion, which paves the way for future scientific studies in this direction. | These changes have been made in the Discussion and Results sections. | Authors agree with the reviewer's comments. |

**FIGURES**

| **Suggested Revisions** | **Revisions Made by Authors** | **Reasoning** |
| --- | --- | --- |
| Figures 1 and 2 are not very helpful. | Figures 1 and 2 have been removed. All other figures have been renumbered. | Removal of Figures 1 and 2 are in response to this comment and other comments about how the Introduction section is too lengthy and that it reads as a “book report.” The authors originally included length as a literature review but understand reviewer suggestions (multiple reviewers said Introduction is too long).  All other figures have been renumbered to account for the deletion of Figures 1 and 2. |
| Figure legends not near figures is cumbersome. Axes labels need explanation. | Figure captions and body text have been updated with clarifying language. | Clarity has been added to avoid confusion. |
| Figures are clear, but NMDS plots could include 95% confidence ellipses. | Captions have been revised to indicate ellipses already present in NMDS plots are 95% confidence ellipses. | Clarity has been added to avoid confusion. |

**ACKNOWLEDGMENTS**

| **Suggested Revisions** | **Revisions Made by Authors** | **Reasoning** |
| --- | --- | --- |
| Thank you for stating the following financial disclosure: “Funding was made possible by a grant from the National Institute of General Medical Sciences (GM103440) from the National Institutes of Health through the Nevada Idea Network of Biomedical Research Excellence (INBRE) grants for both faculty and undergraduate students, Regional Alliance of INBRE Networks (RAIN) Technology Access Grants, and Nevada INBRE Scientific Core Services Awards.”  Please state what role the funders took in the study. If the funders had no role, please state: "The funders had no role in study design, data collection and analysis, decision to publish, or preparation of the manuscript." If this statement is not correct you must amend it as needed. Please include this amended Role of Funder statement in your cover letter; we will change the online submission form on your behalf. | Sentence added to the Cover Letter: “The funders had no role in study design, data collection and analysis, decision to publish, or preparation of the manuscript.” | This statement is true and has been added per editor request. |
| Thank you for stating the following in the Acknowledgments Section of your manuscript: “The authors would like to acknowledge the agencies, labs, and individuals who assisted on this project. Funding was made possible by a grant from the National Institute of General Medical Sciences (GM103440) from the National Institutes of Health through the Nevada Idea Network of Biomedical Research Excellence (INBRE) grants for both faculty and undergraduate students, Regional Alliance of INBRE Networks (RAIN) Technology Access Grants, and Nevada INBRE Scientific Core Services Awards. Genomic sequencing, metabarcoding, and library creation were completed by Idaho State University Molecular Research Core Facility, RRID:SCR_012598 (<https://www.isu.edu/mrcf/>). Statistical analyses and bioinformatics were completed by the Nevada Bioinformatics Center (<https://www.unr.edu/bioinformatics>). Lastly, the authors would like to thank the INBRE summer field students who helped pump liters of water through Sterivex filters.”  We note that you have provided funding information that is not currently declared in your Funding Statement. However, funding information should not appear in the Acknowledgments section or other areas of your manuscript. We will only publish funding information present in the Funding Statement section of the online submission form. Please remove any funding-related text from the manuscript and let us know how you would like to update your Funding Statement. Currently, your Funding Statement reads as follows: “Funding was made possible by a grant from the National Institute of General Medical Sciences (GM103440) from the National Institutes of Health through the Nevada Idea Network of Biomedical Research Excellence (INBRE) grants for both faculty and undergraduate students, Regional Alliance of INBRE Networks (RAIN) Technology Access Grants, and Nevada INBRE Scientific Core Services Awards.”  Please include your amended statements within your cover letter; we will change the online submission form on your behalf. | Funding information has been removed from the manuscript and added to the cover letter. | The author’s completed the requested edit. |

**REFERENCES**

| **Suggested Revisions** | **Revisions Made by Authors** | **Reasoning** |
| --- | --- | --- |
| Some references are too old. All references older than 10 years should be replaced. | The following references have been removed:   - Baldwin (2013) - de Souza et al. (2014) - Griffiths et al. (2008) - Hader et al. (2007) - Hayashi et al. (2012) - Logue et al. (2004) - Quade et al. (1998) - Scarsbrook et al. (2007) - van der Kamp (1995) - Ward (1996) - Winter et al. (2001)   The following references have been kept:   - Apprill et al. (2015) - Austin et al. (2008) - Caporaso et al. (2012) - de Souza et al. (2014) - Hershey (1989) - Kavanaugh (2015) - Page et al. (2005) - Plume (1989) - Quince et al. (2011) - Reuter et al. (2015) - Richardson et al. (2007) - Sada et al. (2005) - Springer and Stevens (2008) - Tingley et al. (2008) - Winograd et al. (1998) | References used as case studies and are 10+ years old have been removed.  Please see below for references that are 10+ years old and remain in the manuscript:   - Apprill et al. (2015) - explains aspects of the protocol used in this study - Austin et al. (2008) - relevant to the manuscript study site (Spring Mountains, NV); not many studies have been done in this geographic region, and the authors deem this study to be relevant to this manuscript. - Caporaso et al. (2012) - explains aspects of the protocol used in this study - Hershey (1989) - thesis relevant to the manuscript study site (Spring Mountains, NV); not many studies have been done in this geographic region, and the authors deem this study to be relevant to this manuscript. - Kavanaugh (2015) - relevant to the manuscript study site (Spring Mountains, NV); not many studies have been done in this geographic region, and the authors deem this study to be relevant to this manuscript. - Page et al. (2005) - most recent geologic map of the study site - Plume (1989) - relevant to the manuscript study site (Spring Mountains, NV); not many studies have been done in this geographic region, and the authors deem this study to be relevant to this manuscript. - Quince et al. (2011) - explains aspects of the protocol used in this study - Reuter et al. (2015) - explains aspects of the protocol used in this study - Richardson et al. (2007) - relevant to the manuscript study site (Spring Mountains, NV); not many studies have been done in this geographic region, and the authors deem this study to be relevant to this manuscript. - Sada et al. (2005) - relevant to the manuscript study site (Spring Mountains, NV); not many studies have been done in this geographic region, and the authors deem this study to be relevant to this manuscript. - Springer and Stevens (2008) - defines springs by morphology; terminology used in this manuscript. - Tingley et al. (2008) - relevant to the manuscript study site (Spring Mountains, NV); not many studies have been done in this geographic region, and the authors deem this study to be relevant to this manuscript. - Winograd et al. (1998) - relevant to the manuscript study site (Spring Mountains, NV); not many studies have been done in this geographic region, and the authors deem this study to be relevant to this manuscript. |
| If the reviewer comments include a recommendation to cite specific previously published works, please review and evaluate these publications to determine whether they are relevant and should be cited. There is no requirement to cite these works unless the editor has indicated otherwise. | No changes made – these references have not been added.  However, other references were added to bolster the Discussion section. | See the explanation below. |
| Suggested References to Add • Li, R., Zhu, G., Lu, S., Meng, G., Chen, L., Wang, Y.,... Wang, Q. (2025). Effects of cascade hydropower stations on hydrologic cycle in Xiying river basin, a runoff in Qilian mountain. Journal of Hydrology, 646, 132342. <https://doi.org/10.1016/j.jhydrol.2024.132342> • Huang, E., Zhu, G., Meng, G., Wang, Y., Chen, L., Miao, Y.,... Li, W. (2025). Historical dataset of reservoir construction in arid regions. Scientific Data, 12(1), 1428. <https://doi.org/10.1038/s41597-025-05712-3> • Yi, Z., Qiu, C., Wang, D., Cai, Z., Yu, J.,... Shi, J. (2024). Submesoscale kinetic energy induced by vertical buoyancy fluxes during the tropical cyclone Haitang. JGR: Oceans, 129(7), e2023JC020494. <https://doi.org/10.1029/2023JC020494> • Li, R., Zhu, G., Chen, L., Qi, X., Lu, S., Meng, G.,... Gun, Y. (2025). Global stable isotope dataset for surface water. Earth Syst. Sci. Data, 17(5), 2135-2145. <https://doi.org/10.5194/essd-17-2135-2025> • Zhang, Y., & Wu, X. (2025). Global space-time patterns of sub-daily extreme precipitation. Environmental Research Letters, 20. <https://doi.org/10.1088/1748-9326/ade607> • D Silva, S., Mathew, B. P., V, K. V., Bhadran, A., Girishbai, D.,... Gopinath, G. (2024). Geomorphic evolution of a tropical river basin. Geology, Ecology, and Landscapes, 1–12. <https://doi.org/10.1080/24749508.2024.2359787> • Abegeja, D., & Nedaw, D. (2024). Identification of groundwater potential zones in Meki Catchment. Geology, Ecology, and Landscapes, 1–16. <https://doi.org/10.1080/24749508.2024.2392380> • Ma, Q., Qian, Y., Su, W., Shi, L., Wang, E., Yu, A.,... Lu, Y. (2025). Degradation of agricultural polyethylene film by greater wax moth larvae. Ecotoxicology and Environmental Safety, 303, 118841. <https://doi.org/10.1016/j.ecoenv.2025.118841> • Sun, S., Xie, W., Wang, G., Zhang, W., Hu, Z., Sun, X.,... DeLuca, T. H. (2025). Evidence for phosphorus cycling parity in N2-fixing pioneer plant species. Functional Ecology, 39(4), 985-1000. <https://doi.org/10.1111/1365-2435.70023> • Zhu, Z., Lu, R., Yu, B., Li, T., & Yeh, S. (2024). A moderator of tropical impacts on climate in Canadian Arctic Archipelago. Nature Communications, 15(1), 8644. <https://doi.org/10.1038/s41467-024-53056-0> | No changes made – these references have not been added.  However, other references were added to bolster the Discussion section. | These references, although useful, are not quite relevant to the current study due to the scale of infrastructure projects (i.e., hydropower and reservoir impacts). Many springs in the southwestern United States are quite small –you could leap across the width of the spring. Spring conservation in the Spring Mountains includes efforts like limiting human and wild ungulate access by constructing fences. Small herds of donkeys, horses, or deer could trample and ruin spring ecosystems, and humans driving off road vehicles heavily alter streambed morphology. Thus, there is a misalignment in stream channel and discharge magnitude when relating these springs to publications discussing hydropower projects. |

**OTHER OVERALL MANUSCRIPT COMMENTS**

| **Suggested Revisions** | **Revisions Made by Authors** | **Reasoning** |
| --- | --- | --- |
| Please ensure that your manuscript meets PLOS ONE's style requirements, including those for file naming. The PLOS ONE style templates can be found at  <https://journals.plos.org/plosone/s/file?id=wjVg/PLOSOne_formatting_sample_main_body.pdf> and  [https://journals.plos.org/plosone/s/file?id=ba62/PLOSOne_formatting_sample_title_authors_affiliations.pdf](https://track.editorialmanager.com/CL0/https:%2F%2Fjournals.plos.org%2Fplosone%2Fs%2Ffile%3Fid=ba62%2FPLOSOne_formatting_sample_title_authors_affiliations.pdf/1/010f0199a4c7fab9-7eadb18f-3fa0-424d-a633-b2bd8ff80011-000000/famIgHoXY7C-6Wmog11BExAWH2AxRQYWWzdBe0WFU5Y=231) | The manuscript meets PLOS ONE’s style requirements. | N/A. |
| We note that you have indicated that there are restrictions to data sharing for this study. PLOS only allows data to be available upon request if there are legal or ethical restrictions on sharing data publicly. For more information on unacceptable data access restrictions, please see <http://journals.plos.org/plosone/s/data-availability#loc-unacceptable-data-access-restrictions>.  Before we proceed with your manuscript, please address the following prompts:  a) If there are ethical or legal restrictions on sharing a de-identified data set, please explain them in detail (e.g., data contain potentially identifying or sensitive patient information, data are owned by a third-party organization, etc.) and who has imposed them (e.g., a Research Ethics Committee or Institutional Review Board, etc.). Please also provide contact information for a data access committee, ethics committee, or other institutional body to which data requests may be sent.  b) If there are no restrictions, please upload the minimal anonymized data set necessary to replicate your study findings to a stable, public repository and provide us with the relevant URLs, DOIs, or accession numbers. For a list of recommended repositories, please see <https://journals.plos.org/plosone/s/recommended-repositories>. You also have the option of uploading the data as Supporting Information files, but we would recommend depositing data directly to a data repository if possible.  We will update your Data Availability statement on your behalf to reflect the information you provide. | Data sharing restrictions have been removed.  Aquatic chemistry data have been added in tables to the Supplemental section.  The authors have not shared the FASTQ genomics data. | Data sharing restrictions have been removed.  FASTQ genomics data have not been uploaded to a repository. The authors would like to ask the editor how to proceed as the authors would like to wait to share these data. Thank you for the consideration. |
| We note that Figure 2b includes an image of a participant. As per the PLOS ONE policy (http://journals.plos.org/plosone/s/submission-guidelines#loc-human-subjects-research) on papers that include identifying, or potentially identifying, information, the individual(s) or parent(s)/guardian(s) must be informed of the terms of the PLOS open-access (CC-BY) license and provide specific permission for publication of these details under the terms of this license. Please download the Consent Form for Publication in a PLOS Journal (http://journals.plos.org/plosone/s/file?id=8ce6/plos-consent-form-english.pdf). The signed consent form should not be submitted with the manuscript, but should be securely filed in the individual's case notes. Please amend the methods section and ethics statement of the manuscript to explicitly state that the patient/participant has provided consent for publication: “The individual in this manuscript has given written informed consent (as outlined in PLOS consent form) to publish these case details”.  Please respond by return e-mail with an amended manuscript. We can upload this to your submission on your behalf.  If you are unable to obtain consent from the subject of the photograph, please either instruct us to remove the figure or supply a replacement figure by return e-mail for which you hold the relevant copyright permissions and subject consents. In some cases, you may need to specify in the text that the image used in the figure is not the original image used in the study, but a similar image used for illustrative purposes only. We can make any changes on your behalf. | No consent needed as Figure 2b has been removed. | Removal of Figures 1 and 2 are in response to this comment and other comments about how the Introduction section is too lengthy and that it reads as a “book report.” The authors originally included length as a literature review, but understand reviewer suggestions (multiple reviewers said Introduction is too long). |
| This manuscript is not ready for publication in a scientific journal. The report appears as an undergraduate project and reads that way. Although the topic of bacteria in oligotrophic waters can be of importance and interest, this project appeared to be the training of students in both engaging students in field work as well as training them on bioinformatic and eDNA, and less about sound hypothesis development and robust science to answer the questions. Data are not yet available (see front matter). | Authors have made proper changes and believe this manuscript is ready for publication in a scientific journal. | Authors have made proper changes and believe this manuscript is ready for publication in a scientific journal. |
| The manuscript is well-written, methodologically sound, and provides valuable insights into the chemistry and microbial community composition of cold-water springs in the Spring Mountains. The integration of environmental DNA (eDNA) with aquatic chemistry strengthens the work and fills a regional knowledge gap. The findings on elevation, disturbance, and microbial diversity are novel and important for conservation and water management in arid landscapes. | The authors appreciate this comment and have also made the proper changes. | The authors appreciate this comment and have also made the proper changes. |
| Minor Revision – The study is publishable after addressing the contextual gaps in the introduction/discussion and incorporating broader hydrological and climatic perspectives. | The authors have made proper changes. | The authors have made proper changes. |
